# Supplementary material for: Drug repurposing for aging research using model organisms
Source: Aging Cell. 2017 Jun 16;16(5):1006–15. doi: 10.1111/acel.12626 (PMC5595691; doi:10.1111/acel.12626)
Supplement: Supplementary file 7 — Data S1 Zip‐Archive of all report cards. [file ACEL-16-1006-s007.zip › RC_P16.pdf]

P16

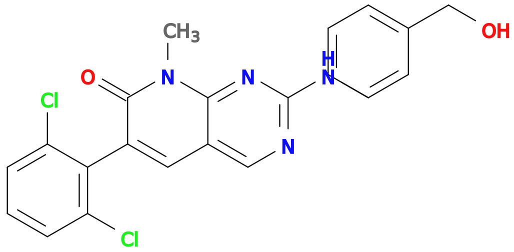

Database identifiers

ChEMBLCompound	CHEMBL327127
DrugBank	DB08339
eMolecules	36796303

Ranking

	Rank	Score
Drosophila	419/697	0.342
C. elegans	245/591	0.224

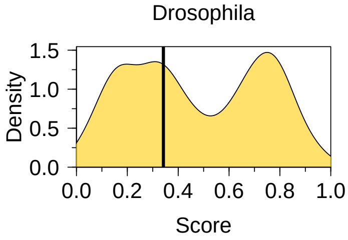

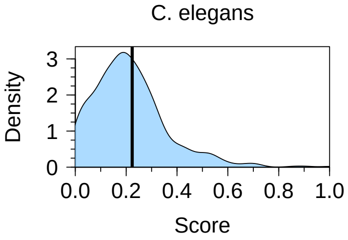

	Ageing implication	Domain conservation	Binding site conservation	Binding affinity	Bioavailability	Lipinski	Promiscuity	Purchasability	Drug approval	**Total**
Drosophila	0.203	0.966	1.0	0.95	(0.9)	0.0	-0.0	0.1	0.075	0.342
C. elegans	0.203	0.952	1.0	0.95	0.268	0.0	-0.0	0.1	0.075	0.224

Names

- 2-(3-(hydroxymethyl)phenylamino)-6-(2,6-dichlorophenyl)-8-me thylpyrido2,3-dpyrimidin-7(8H)-one
- 6-(2,6-dichlorophenyl)-2-3-(hydroxymethyl)phenylamino-8-methyl-pyrido6,5-dpyrimidin-7-one
- 6-(2,6-dichlorophenyl)-2-3-(hydroxymethyl)phenylamino-8-methylpyrido2,3-dpyrimidin-7(8h)-one
- 6-(2,6-dichlorophenyl)-8-methyl-2-(3-methylolphenyl)aminopyrido6,5-dpyrimidin-7-one

Roles

ChEBI entry None has no roles

Status

Approved drug (according to ChEMBL)	No
Classification (according to DrugBank)	experimental
Number of Rule of 5 violations	0
Binding affinity to original target in log units (RF-Score prediction)	7.95
Burns *C. elegans* bioavailability prediction	-1.33

Compound Target Characteristics

Tyrosine-protein kinase ABL1

Best gene implication in ageing for this target family came from gene P00519 via mapping the annotation from Ensembl ENSG00000097007 via mapping the annotation from EntrezGene 25 via mapping the annotation from GenAgeHuman 0078 annotated in GenAge release 17.

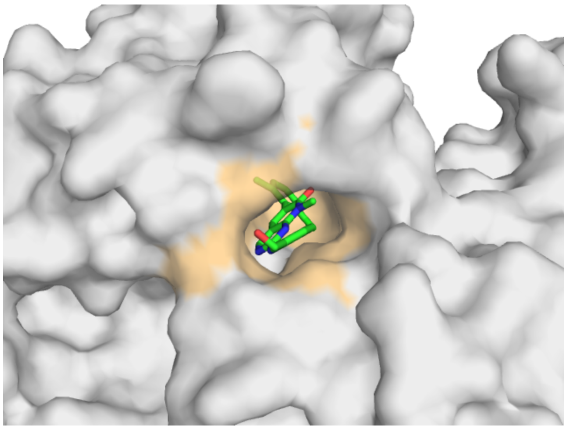

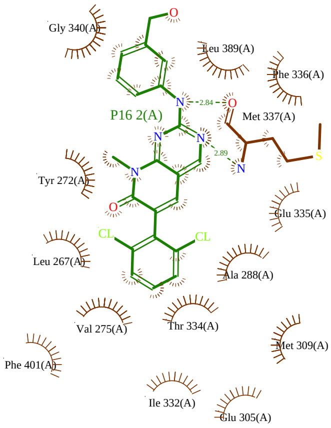

protein	amino acids contacts (binding site)
PDB:1opk:chainA:P00520	L Y V A K E M I T E F M G L A D F
sp:P00519:ABL1_HUMAN	L Y V A K E M I T E F M G L A D F
tr:Q59FK4:Q59FK4_HUMAN	L Y V A K E M I T E F M G L A D F
tr:F1M0A6:F1M0A6_RAT	L Y V A K E M I T E F M G L A D F
tr:D3ZGM3:D3ZGM3_RAT	L Y V A K E M I T E F M G L A D F
tr:E9PT20:E9PT20_RAT	L Y V A K E M I T E F M G L A D F
tr:Q3V2W1:Q3V2W1_MOUSE	L Y V A K E M I T E F M G L A D F
sp:P00520:ABL1_MOUSE	L Y V A K E M I T E F M G L A D F
tr:Q3SYK5:Q3SYK5_MOUSE	L Y V A K E M I T E F M G L A D F
sp:P00522:ABL_DROME	L Y V A K E M I T E F M G L A D F
tr:A8JNU2:A8JNU2_DROME	L Y V A K E M I T E F M G L A D F
sp:P03949:ABL1_CAEEL	L Y V A K E M I T E F M G L A D F

	whole protein		domain-based		contact-based	
protein	ident	simil	ident	simil	ident	simil
PDB:1opk:chainA:P00520	1.0	1.0	1.0	1.0	1.0	1.0
sp:P00519:ABL1_HUMAN	0.88	0.95	1.0	1.0	1.0	1.0
tr:Q59FK4:Q59FK4_HUMAN	0.83	0.92	1.0	1.0	1.0	1.0
tr:F1M0A6:F1M0A6_RAT	0.93	0.96	1.0	1.0	1.0	1.0
tr:D3ZGM3:D3ZGM3_RAT	0.96	0.99	1.0	1.0	1.0	1.0
tr:E9PT20:E9PT20_RAT	0.93	0.96	1.0	1.0	1.0	1.0
tr:Q3V2W1:Q3V2W1_MOUSE	0.33	0.33	1.0	1.0	1.0	1.0
sp:P00520:ABL1_MOUSE	1.0	1.0	1.0	1.0	1.0	1.0
tr:Q3SYK5:Q3SYK5_MOUSE	0.96	0.98	1.0	1.0	1.0	1.0
sp:P00522:ABL_DROME	0.29	0.51	0.78	0.93	1.0	1.0
tr:A8JNU2:A8JNU2_DROME	0.29	0.5	0.78	0.93	1.0	1.0
sp:P03949:ABL1_CAEEL	0.22	0.43	0.67	0.9	1.0	1.0

Abl (FBgn0000017) associated phenotypes

cell migration defective, cell shape defective, germline clone, heat sensitive, lethal - all die before end of pupal stage, maternal effect, mitotic cell cycle defective, neuroanatomy defective, neurophysiology defective, partially lethal - majority die, partially lethal - majority live, reduced, rescuable maternal effect, size defective, somatic clone, some die during pupal stage

(Information from FlyBase)

Abl (UniProt:P00522) annotation

Function: Arm and Abl proteins function cooperatively at adherens junctions in both the CNS

and epidermis; critical for embryonic epithelial morphogenesis regulating cell shape changes and cell migration. Plays a critical role in transducing embryonic midline repulsive cues; may regulate cytoskeletal dynamics underlying a growth cone's response to midline cues. The ability of pCC/MP2 axons to correctly interpret midline repulsive cues and stay on the ipsilateral side is dependent on the strength of both Slit/robo and Abl-dependent signaling pathways. (PubMed:11756472, PubMed:12973825, PubMed:9635189).
Subcellular location: Cytoplasm.
Tissue specificity: Arm and ena colocalize with Abl at adherens junctions throughout development. (PubMed:11756472, PubMed:9635189).
Developmental stage: Expressed both maternally and zygotically. (PubMed:2832740).
Disruption phenotype: Both loss- and gain-of-function mutants exhibit neurons within the pCC/MP2 pathway that incorrectly project across the midline. Loss of Abl disrupts cell migration and cell shape changes during dorsal closure. (PubMed:11756472, PubMed:9635189).

(Information from UniProt)

abl-1 (WBGene00000018) associated phenotypes

apoptosis variant, gamma ray induced apoptosis increased, lipid synthesis increased, protein subcellular localization variant

(Information from WormBase)
